# Leveraging Fecal Bacterial Survey Data to Predict Colorectal Tumors

**DOI:** 10.3389/fgene.2019.00447

**Published:** 2019-05-28

**Authors:** Bangzhou Zhang, Shuangbin Xu, Wei Xu, Qiongyun Chen, Zhangran Chen, Changsheng Yan, Yanyun Fan, Huangkai Zhang, Qi Liu, Jie Yang, Jinfeng Yang, Chuanxing Xiao, Hongzhi Xu, Jianlin Ren

**Affiliations:** ^1^Department of Gastroenterology, Zhongshan Hospital Xiamen University, Xiamen, China; ^2^Institute for Microbial Ecology, School of Medicine, Xiamen University, Xiamen, China; ^3^Xiamen Treatgut Biotechnology Co., Ltd., Xiamen, China; ^4^Department of Gastroenterology, The Affiliated Hospital of Guizhou Medical University, Guiyang, China

**Keywords:** fecal bacteria, colorectal cancer, colorectal adenoma, random forest, random effects model

## Abstract

Colorectal cancer (CRC) ranks second in cancer-associated mortality and third in the incidence worldwide. Most of CRC follow adenoma-carcinoma sequence, and have more than 90% chance of survival if diagnosed at early stage. But the recommended screening by colonoscopy is invasive, expensive, and poorly adhered to. Recently, several studies reported that the fecal bacteria might provide non-invasive biomarkers for CRC and precancerous tumors. Therefore, we collected and uniformly re-analyzed these published fecal 16S rDNA sequencing datasets to verify the association and identify biomarkers to classify and predict colorectal tumors by random forest method. A total of 1674 samples (330 CRC, 357 advanced adenoma, 141 adenoma, and 846 control) from 7 studies were analyzed in this study. By random effects model and fixed effects model, we observed significant differences in alpha-diversity and beta-diversity between individuals with CRC and the normal colon, but not between adenoma and the normal. We identified various bacterial genera with significant odds ratios for colorectal tumors at different stages. Through building random forest model with 10-fold cross-validation as well as new test datasets, we classified individuals with CRC, advanced adenoma, adenoma and normal colon. All approaches obtained comparable performance at entire OTU level, entire genus level, and the common genus level as measured using AUC. When combined all samples, the AUC of random forest model based on 12 common genera reached 0.846 for CRC, although the predication performed poorly for advance adenoma and adenoma.

## Introduction

Colorectal cancer (CRC) ranks second in term of cancer-associated mortality and third in term of incidence, with an estimation of 881000 deaths and over 1.8 million new cases in 2018 in both sexes globally (Bray et al., 2018). CRC incidence rates are about 3-fold higher in developed countries than developing ones. The incidence and mortality rates also showed an increasing trend in China in the past decades. The age-standardized incidence and mortality rates by world standard population are 17.52 and 7.91 per 100000 in 2014, respectively (Chen W. et al., 2018). Survival exceeds 90% if the cancer is detected at early stage, but decreases to 13% with advanced metastatic disease (Shah et al., 2018). Moreover, development of most CRC cases follows adenoma-carcinoma sequence, spanning more than 10–15 years in average. Therefore, targeting the CRC by early screening and treatment, especially as early to the adenoma stage, would have profound clinical and socioeconomic significances.

Colonoscopy is regarded as the golden standard of CRC screening. However, this test is poorly adhered to due to the invasiveness, frequency, and expensive price. For example, it is reported that more than 25% of adults aged 50–75 years, the high-risk group, never participated for CRC screening in United States (Centers for Disease Control and Prevention, 2018). A recent survey in China showed a more serious screening situation, only 14% of high risk people evaluated by a score system finally undertaking colonoscopy screening (Chen H. et al., 2018). Home-based fecal occult blood tests (FOBT) have low sensitivity in colorectal adenoma (CRA) or pre-cancers (Hundt et al., 2009), and are used less frequently. Thus, development of non-invasive and sensitive early diagnosis tests for CRC or precancerous lesions are in urgent need for improving the patient participation rate.

In the past years, numerous studies using mouse models or case-control designs have shown the effects of both individual gut microbes (Goodwin et al., 2011; Rubinstein et al., 2013; Abed et al., 2016) and the overall community (Baxter et al., 2014; Zackular et al., 2016) in disease progression of CRA and CRC. The roles of gut microbiota hypothesized in tumorigenesis, acting as environmental factors, also accord with the sporadic nature of CRC and CRA. Therefore, extensive efforts have been put into identify microbiota-associated biomarkers for colorectal tumors (Ahn et al., 2013; Zeller et al., 2014; Baxter et al., 2016; Yu et al., 2017; Flemer et al., 2018). Although some taxa, including Fusobacterium, Peptostreptococcus, and Porphyromonas, were consistently reported to be enriched in CRC, unifying signal taxa were not defined. Moreover, most studies focused on CRC, but attention to CRA is factually in great clinical need to facilitate early detection of the tumors. Recently, there were two meta-analyses based on 16S rRNA gene sequences, which were helpful for distilling possible biomarkers and classifying patients with adenoma or carcinoma. However, the aggregate number of samples was smaller (*n* = 509) (Shah et al., 2018), and sequencing depths of some studies included were quite low (Shah et al., 2018; Sze and Schloss, 2018). Furthermore, several case-control studies with higher depths have been reported since the publication of these two meta-analyses. Therefore, it is meaningful and urgent to update the analysis to facilitate the development of non-invasive diagnosis tests for colorectal tumors based on fecal microbiota.

In this study, we updated meta-analysis using fecal 16S rRNA gene sequence data from 7 studies with a relatively higher sequencing depth (more than 5000 reads/sample). By the most frequently used methods, we sought to determine the bacterial variation among studies, the differences in fecal bacteria diversity and communities in patients with colorectal tumors, and identify a universal set of microbial markers to predict/diagnose the presence of colorectal cancer.

## Materials and Methods

### Datasets

The studies included in this meta-analysis were screened from two sources: systematic Pubmed search with colorectal (colon) cancer (CRC) or adenoma (CRA) and gut microbiota in the past 10 years, and the recently published reviews and meta-analyses. Studies were excluded if (1) samples were not from feces, (2) samples were not sequenced by NGS for 16S rRNA gene, (3) sequences, barcodes, or metadata were not publicly available or not provided by authors until Sep 20, 2018 after requests by emails, (4) the sequencing depth was lower than 5000 raw reads. At last, we obtained sequence datasets and metadata from 7 studies with CRC and/or CRA (Zeller et al., 2014; Baxter et al., 2016; Flemer et al., 2017; Hale et al., 2017; Deng et al., 2018; Flemer et al., 2018; Mori et al., 2018), additional 12 studies associated gut microbiota of colorectal lesions were excluded due to lower sequencing depth, incomplete information of sequences, barcodes, or metadata (Sobhani et al., 2011; Chen et al., 2012; Wang et al., 2012; Ahn et al., 2013; Brim et al., 2013; Chen et al., 2013; Weir et al., 2013; Wu et al., 2013; Goedert et al., 2015; Mira-Pascual et al., 2015; Ai et al., 2017; Zhang et al., 2018). In summary, all 7 studies had CRC samples, 4 studies had advanced adenoma (Adv_adenoma, >10 mm in size) samples, and 4 studies had samples with adenoma smaller than 10 mm (Table 1).

**Table 1:** characteristics of the fecal 16S rDNA sequencing studies included in the meta-analysis.

No.	Author, year	Country	Source^∗^	Health	Polyps	Adenoma (<1 cm)	Adv_adenoma (>1 cm)	CRC	DNA extraction	Region	Seq platform
1	Deng et al., 2018	China	SRA	33	0	0	0	17	GenElute Stool DNA isolation Kit	V3-V4	HiSeq
2	Flemer et al., 2018	Ireland	Author	62	0	22	0	69	Allprep DNA/RNA kit-Qiagen	V3-V4	MiSeq
3	Mori et al., 2018	Italy	Author	18	14	18	21	8	QIAamp DNA stool kit	V4	Miseq
4	Flemer et al., 2017	Ireland	Author	36	0	0	0	42	Allprep DNA/RNA kit-Qiagen	V3-V4	MiSeq
5	Hale et al., 2017	United States	Author	475	0	0	203	34	Chemagic DNA Blood Special Kit	V3-V5	MiSeq
6	Baxter et al., 2016	United States + Canada	SRA	172	0	88	108	119	PowerSoil	V4	MiSeq
7	Zeller et al., 2014	France	SRA	50	0	13	25	41	GNOME DNA Isolation Kit(MP)	V4	Miseq
8	Zhang et al., 2018	China	NA	130	30	32	88	130	OMEGA-soil DNA kit	V3-V4	MiSeq
9	Ai et al., 2017	China	NA	52	0	47		42	E.Z.N.A. Stool DNA Kit	V1-V3	454
10	Goedert et al., 2015	China	NA	24	9	0	20	2	–	V3-V4	MiSeq
11	Mira-Pascual et al., 2015	Spain	NA	10	0	11		7	Macherey–Nagel	V1-V3	454
12	Ahn et al., 2013	United States	NA	94	0	0	0	47	PowerSoil	V3-V4	454
13	Brim et al., 2013	United States	SRA	6	6	0	0	0	QIAamp Stool DNA	V1-V3	454
14	Chen et al., 2013	China	NA	47	0	0	47	0	Bead beating methods and phenol-chloroform	V1-V3	454
15	Weir et al., 2013	United States	NA	8	0	0	0	7	MoBio Powersoil	V4	454
16	Wu et al., 2013	China	NA	20	0	0	0	19	QIAamp Stool DNA	V3	454
17	Chen et al., 2012	China	NA	21	0	0	0	22	QIAamp DNA Mini Kit	V1-V3	454
18	Wang et al., 2012	China	NA	56	0	0	0	46	Bead-beating extraction and phenol–chloroform	V3	454
19	Sobhani et al., 2011	France	NA	6	0	0	0	6	GNOME DNA Isolation Kit(MP)	V3-V4	454

*^∗^NA indicates studies were not included in the analysis, either due to the datasets not available, without barcode sequences to splits the datasets, or low sequencing depth/sample.*

### Sequence Processing

Paired-end reads were assembled using FLASH by default parameters, except with -x 0.2 and -M 200 for V3-V4 /-M 250 for V3-V5 /-M 150 for V4 region. The assembled sequences were quality filtered with a minimum quality score of 25. To assign *de novo* OTUs, we removed chimeric sequences and clustered sequences with 97% similarity and using Usearch (Edgar, 2013) for individual study. The representative sequences of OTUs were aligned to the SILVA database for taxonomic classification by RDP Classifier (Wang et al., 2007) and aggregate to various taxonomic levels.

### Community Analyses

The alpha-diversity metrics, including observed OTUs (Obs), Shannon, and Pielou’s evenness (J), were calculated based on OTU table evenly rarefied to the lowest sequencing depth within each study. The differences between individual with normal colon, adenoma, or CRC were further tested by Wilcoxon test for significance. We also calculated the ORs of these metrics by assigning any value above the median of the metric within the study as positive. The beta diversity based on Bray-Curtis distance was measured within each study, and the differences between groups were determined using permutational analysis of variance (PERMANOVA) with 9999 permutations. In terms of genera, the differences between groups were examined using Wilcoxon test within each study, and the ORs were determined in the same manner as alpha diversity metrics. Finally, both random effects (RE) model and fixed effects (FE) model were used to obtain the change summary estimates.

### Classification by Random Forest

To estimate the predictive power of gut microbiota for classifying individuals with normal colon and colorectal tumors, the most widely used and robust random forest models were selected and built for each study based on all OTUs, all genera, and the common genera that were detected in every study. RF model based on all studies and n-1 (leave-one-study-out) studies were also built to further assess the classifier performance of the common genera and the weight of particular study to the overall performance, respectively. To test the generalizability, we built RF model based on the common genera from one study and validated it in the other studies, and also performed leave-one-study-out analyses by setting the study left out as the test dataset. All the models were built using a 10-fold cross-validation with ten repeats and the number of features (mtry) was set to the square root of total number of microbial features.

### Statistical Analyses and Visualization

All statistical analyses were conducted in R-3.4.1 (R Core Team, 2017). The alpha-diversity metrics, Bray-Curtis distances by vegdist function, and PERMANOVA by adonis function were all performed in vegan (Oksanen et al., 2015). The ORs were analyzed using epiR (Stevenson et al., 2018) and meta for (Viechtbauer, 2017) with significance testing utilized the chi-square test. In addition, the RF, SVM, KNN, and Adaboost models were built using caret (Kuhn et al., 2017) and random Forest (Breiman et al., 2015) by default parameters, and the test cohorts were predicted using the pROC (Robin et al., 2017). The random effects model and fixed effects model were conducted in metaphor (Viechtbauer, 2017). All figures were plotted using ggplot2-v3.0.0 (Wickham et al., 2017) and gridExtra (Auguie and Antonov, 2016).

## Results

### Sample Variation

**FIGURE 1:**
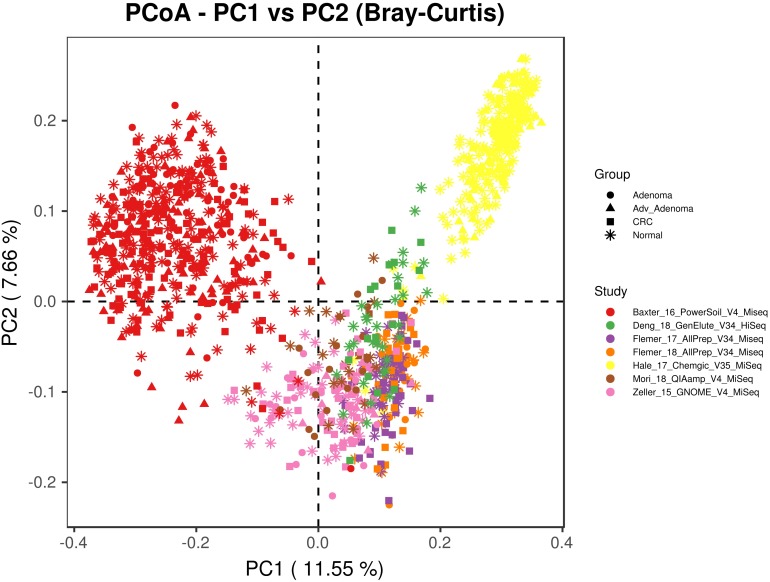
The principal coordinates analysis depicting the great microbial variations from different studies with variables of DNA extraction methods, PCR amplification conditions, sequencing platforms, etc. The points represent samples, shapes represent the different diagnosis, and the colors represent the different study.

We included 16S rRNA gene sequencing data from 7 fecal studies with diseases of CRC, adv_adenoma and adenoma (Table 1). A total of 1674 samples from 7 countries were retained after quality filtering, including 330 CRC, 357 Adv_adenomas, 141 adenoma, and 846 controls. At the beginning, we tried to combine all samples together by closed_reference OTU assignment strategy for compatibility with differential sequencing regions, but found samples clustered primarily by individual studies due to the extra strong variables of DNA extraction methods, PCR amplification conditions, sequencing platforms adopted by individual study (Figure 1). Therefore, we processed each study separately using the same parameters in the following analyses.

### Alpha-Diversity Differences

**FIGURE 2:**
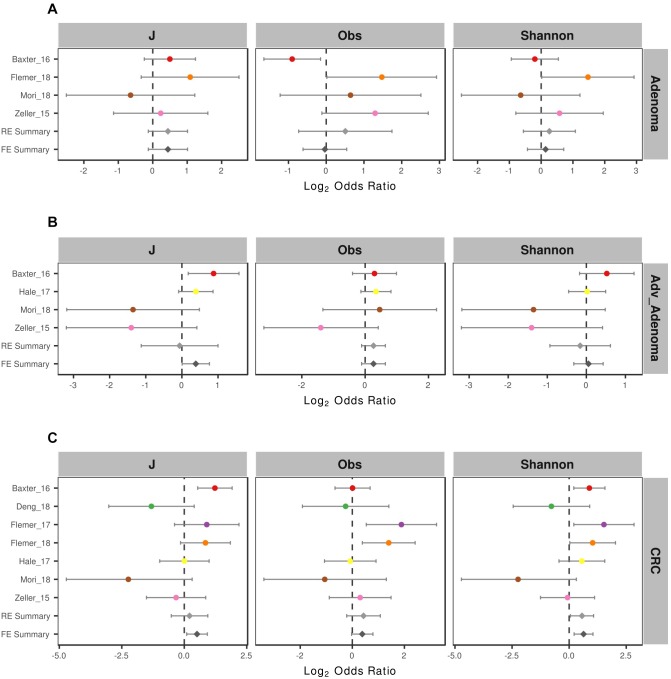
Forest plot of the alpha diversity metrics for **(A)** adenoma, **(B)** advance adenoma, and **(C)** colorectal cancer. The length of the error bar represents the 95% confidence interval. The left of dashed lines depicts that the metric of the case is higher than the control. And the right of dashed lines depicts that the metric of the case is lower than the control. It shows that there were significantly difference between the cases and the control, if there was no overlap between the dashed lines and the error bar.

To compare the alpha-diversity between different disease stages, we considered the microbial richness (Observed OTUs, Obs), Shannon diversity, and evenness J. We found significant higher richness and Shannon diversity in normal colon than CRC in 2 of 7 studies and significant higher microbial evenness in normal colon in 1 of 7 studies (Supplementary Table S1). For comparisons in adenoma vs. normal colon and adv_adenoma vs. normal colon, only one study was significantly different among the richness and evenness. Due to the inconsistent results, we also calculated the odds ratios (ORs). The ORs for Shannon diversity were significantly higher than 1.0 for CRC (OR = 1.48, CI in 1.04 to 2.10) (Figure 2) in both RE model and FE model with low heterogeneity (Supplementary Table S1), indicating significant lower microbial Shannon diversity in CRC than the normal colon group. While The ORs for J, Obs, and Shannon were not significantly greater than 1.0 for adenoma and adv_adenoma in the random effects model with higher heterogeneity, even with the trend (Figure 2).

### Beta-Diversity Differences

**FIGURE 3:**
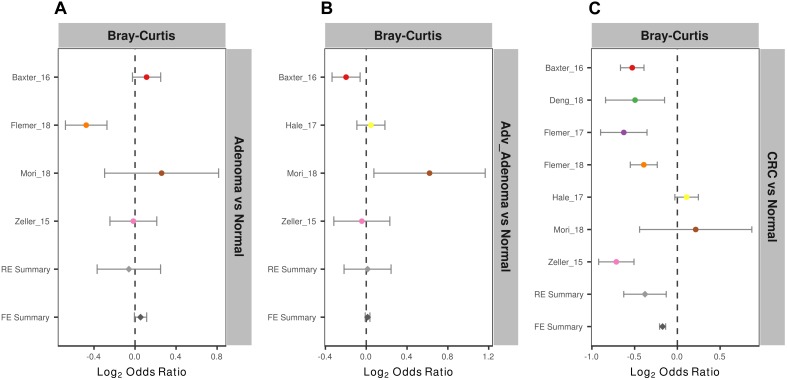
Forest plot of the Bray-Cutris distances between the individual with colorectal tumors and the normal colons. **(A)** Adenomas vs. normal colons; **(B)** Adv_adenomas vs. normal colons; **(C)** CRC vs. normal colons. The error bar depicts the 95% confidence interval. The left-hand side (minus value) of the dashed line depicts that distances between the case and the normal are higher than the distances between the subjects of control. The right-hand side of the dashed line depicts that distances between the case and the normal are lower than the distances between the control. There were significantly difference between the case and the control, if there was no cross between the dashed line and the error bar.

To measure the entire community differences between different individuals with colorectal tumors and with normal colon, we calculated a Bray-Curtis distance matrix for each data set and tested the significance by PERMANOVA. We found significantly different community structure in the CRC relative to normal colons in 6 of 7 studies (Supplementary Table S2 and Supplementary Figure S1). However, we only found significant community differences in adv_adenoma vs. normal in 1 of 4 studies and in adenoma vs. normal in 1 of 4 studies. Again, by calculating the ORs based on the Bray-Curtis metric in each study, we found the significant bacterial community differences between CRC and normal colons in both RE models with high heterogeneity (Supplementary Table S2), but not significant differences in comparisons of adv_adenoma or adenoma with individuals with normal colons (Figure 3). These results showed that there were dependable and significant community-wide changes in the bacterial community structures of CRC patients.

### Different Taxa

With the altered overall community differences, we tried to identify the significantly different taxa between subjects with colorectal tumors and the normal. However, the results were not consistent by Wilcoxon tests (Supplementary Tables S3–S5). By quantifying the ORs, a total of 13 genera were identified to be associated with CRC (Supplementary Figure S2). Five genera had significant ORs lower than 1.0 for presence of CRC in RE and FE models (Supplementary Table S6), including F*usobacterium*, *Lachnospiraceae_UCG-010, Mogibacterium, Oscillibacter, Prevotella_7*. Eight genera possessed significant ORs higher than 1.0 for the absence of CRC, most of which were thought to be beneficial for butyrate production in intestines, including *Anaerostipes*, *Butyricicoccus*, *Coprococcus_2*, *Roseburia*. Besides, a total of 10 genera had significant ORs for the adenoma, and 6 genera had significant ORs for adv_adenoma.

### Development of Fecal Bacteria-Based Classifier

Since the gut microbial communities were greatly shifted with colorectal tumors, especially in CRC compared to the normal, it is meaningful and profound to identify microbial biomarkers for development of invasive diagnosis methods. With this purpose, – we built RF models based on OTU abundance (finer-level) and genus abundance (more general) to classify/predict colorectal tumor and controls within each study.

**FIGURE 4:**
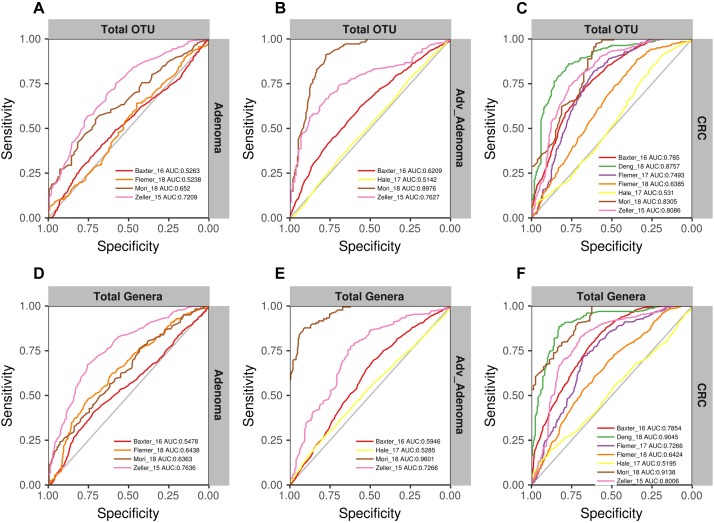
The ROC curves of the each study based on the matrix of the total OTUs **(A–C)** and the matrix of the total genera **(D–F)**. The gray lines represent the random predictors. The other lines depict the ROC curves of each study using the cross-validation with ten repeats.

We found that the RF models using all OTUs did a good job in classifying CRC and individuals with normal colons [median AUC = 0.765, ranging in (0.531, 0.8757)] (Figure 4C). As expected, the RF models based on the genera also showed comparable performance in differentiating CRC and the normal [median AUC = 0.755, ranging in [0.533, 0.977)] (Figure 4F). However, the performances of RF models differentiating adv_adenoma or adenoma and the normal colons were unsatisfactory, just a slightly better than the random predictor in both OTU level [adv_adenoma: median AUC = 0.568, ranging in (0.514, 0.898), adenoma: median AUC = 0.589, ranging in (0.524, 0.721)] (Figure 4A,B) and genus level [adv_adenoma: median AUC = 0.650, ranging in (0.515, 0.99); adenoma: median AUC = 0.598, ranging in (0.515, 0.650)] (Figure 4D,E).

**FIGURE 5:**
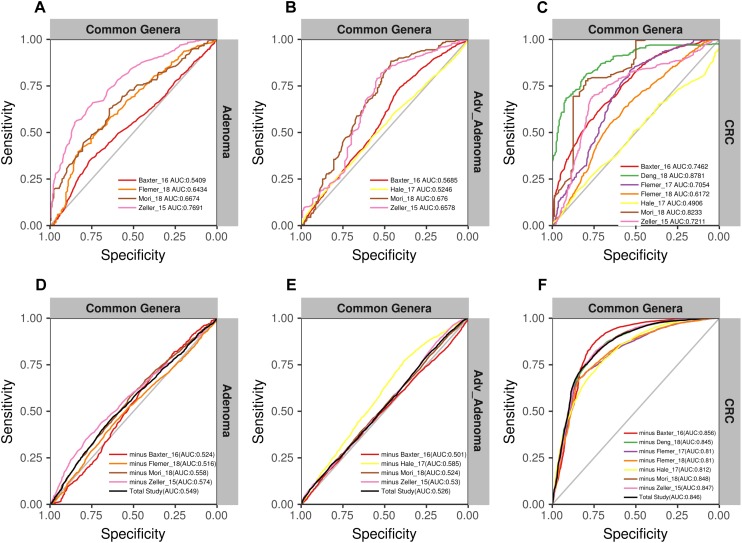
The ROC curves based on the matrix of the common genera. The gray lines represent the random predictors. The other lines depict the ROC curve of each study using the cross-validation with ten repeats **(A–C)**. The black lines represent the curves of the models built using the total studies data sets with cross-validation, and the colorful lines represent the curves of the models using the combined studies data sets with minus a specific study **(D–F)**.

Due to the separate clustering for each study, the above RF models based on all OTUs and all genera were not universal for each other. Therefore, we tried to build the models based on the common genera that detected in every study. Surprisingly, the performance of the models for distinguishing the CRC and individuals with normal colons were good [median AUC = 0.735, ranging in (0.5258, 0.888)] (Figure 5C), while the models for adv_adenoma or adenoma were still weak [adv_adenoma: median AUC = 0.632, ranging in (0.520, 0.693); adenoma: median AUC = 0.603, ranging in (0.521, 0.700)] (Figure 5A,B). When combined all samples and all studies together, RF model returned an AUC of 0.835 for CRC vs. the normal (Figure 5F), which is better than the medium AUC of RF models based on single study, although the prediction of Adv_adenoma or adenoma with the normal was still not good (Figure 5D,E). To test whether particular study weight the performance, we re-built RF models based on n-1 studies (leave-one-study-out), and found the performances were not affected too much (Figure 5D–F), indicating the stability of RF model for CRC based on all 7 studies and the common genera.

**FIGURE 6:**
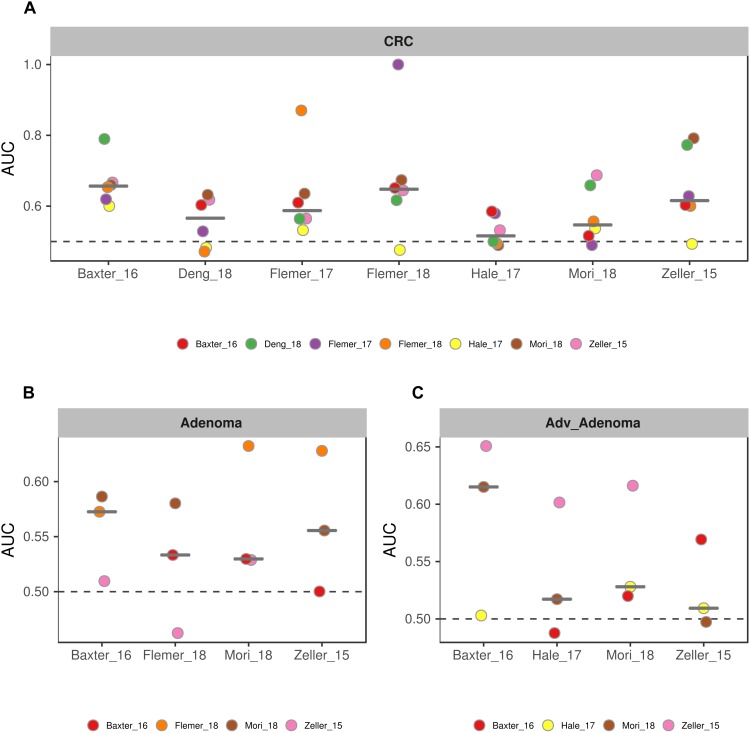
The performances of models to classify the case and the normal. **(A)** CRC vs. normal colons; **(B)** Adenoma vs. normal colons; **(C)** Adv_adenoma vs. normal colons. The horizontal ordinates depict the studies used as the training data set. The vertical coordinates depicts the AUC of the specific test study. The black line represent the median of AUC of all test AUCs for a specific model. The dashed gray lines represent the AUC at 0.5 with random predictors.

**FIGURE 7:**
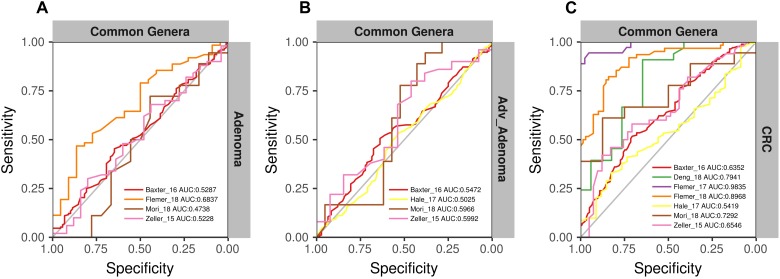
The ROC curves of the models built using the matrix of the common genera and n-1 studies (leave-one-study-out) and validated in the specific study. **(A)** Adenoma vs. normal colons; **(B)** Adv_Adenoma vs. normal colons; **(C)** CRC vs. normal colons.

To further test the generalizability of models based on common genera, we evaluated how well the models would perform when given data from a different cohort. First, we used one study as training data and the other single studies as test data. We found that the performances of the models were different among the training cohorts, probably associated with the sample size (Figure 6). In addition, the performances of the models for CRC were better than the adv_adenoma and adenoma. Within Adv_adenoma, models based on studies of Baxter_16 and Hale_17 were better than other two (Figure 6C). Second, we tested the leave-one-study-out analysis again. As expected, the performances of models were still good for CRC [median AUC = 0.754, ranging in (0.569, 0.916)] (Figure 7C), even still weak for adv_adenoma [median AUC = 0.550, ranging in (0.496, 0.578)] and for adenoma [median AUC = 0.539, rang in (0.494, 0.684)] (Figure 7A,B).

### Important Microbial Taxa as Potential Biomarkers

By looking deeper into the microbial features selected for the RF model for CRC based on all studies, we obtained the 12 important distinguishing taxa based on the mean decrease Gini value (Table 2). Indeed, all these genera were frequently detected in human fecal samples and were previously reported to be harmful to human health, such as the *Fusobacterium*, *Escherichia_Shigella*, and *Streptococcus* with higher abundance in CRC group. Besides, some genera selected by RF model were found to be beneficial with higher abundance in individuals with normal colons, including *Bifidobacterium, Lachnospira.* Furthermore, 4 genera were also overlapped with the significant OR taxa by RE model. In short, the microbial features selected for RF model coincided with their abundance and might reflect their physiological effects.

**Table 2:** Importance, odd ration, heterogeneity, and relative abundance of the 9 common genera selected for the RF model for CRC based on all samples.

Genera	Mean decrease Gini	Odd ratio	CI_lb	CI_ub	*P*-value	*I*^2^	Abundance (%) in CRC	Abundance (%) in the normal
*Bifidobacterium*	15.72	1.34	0.85	2.12	0.2	36.78	1.087 ± 1.019	1.23 ± 0.87
*[Eubacterium]_hallii_group*	13.43	1.76	1.17	2.65	0.01	27.3	0.979 ± 0.846	1.417 ± 1.024
*Streptococcus*	12.9	1.17	0.87	1.58	0.31	0	1.365 ± 0.726	1.136 ± 1.239
*Fusobacterium*	10.75	0.34	0.24	0.48	0	0	0.791 ± 1.444	0.106 ± 0.125
*Escherichia.Shigella*	10.13	0.63	0.37	1.09	0.1	61.3	2.565 ± 1.431	1.368 ± 0.898
*Akkermansia*	8.7	1.01	0.69	1.47	0.97	21.65	1.915 ± 1.681	2.055 ± 1.769
*Lachnospira*	8.11	1.65	1.14	2.4	0.01	31.98	0.379 ± 0.382	0.509 ± 0.376
*Faecalibacterium*	8.09	1.01	0.63	1.63	0.95	55.87	6.69 ± 3.042	6.624 ± 2.294
*un_f__Lachnospiraceae*	7.54	1.48	1.03	2.11	0.03	27.84	1.406 ± 0.968	1.703 ± 1.307
*Prevotella_7*	6.86	0.54	0.38	0.75	0	0	0.522 ± 0.421	0.169 ± 0.129
*Roseburia*	6.78	1.59	1.2	2.12	0	0	1.122 ± 0.329	1.418 ± 0.564
*Lachnospiraceae_UCG.010*	6.62	0.65	0.49	0.87	0	0	0.256 ± 0.209	0.093 ± 0.046

*CI_lb, confidence interval_lower bound; CI_ub, confidence interval_upper bound; I2, heterogeneity measure.*

## Discussion

In this study, we conducted a comprehensive meta-analysis on a diverse collection of 16S rDNA sequencing studies with relatively higher sequencing depth from 6 countries to reveal the great differences in fecal bacterial communities in individuals with colorectal tumors and normal colons. By analyzing all datasets in a uniform manner, we further identified and validated fecal bacterial biomarkers and their important roles in classifying subjects with colorectal tumors, especially the CRC and the normal control. The good performance of common bacterial genera-based RF model demonstrated the great clinical significance and feasibility of development of invasive screening or diagnosis method for CRC by detection of fecal bacterial communities.

Although there were great heterogeneity associated with each original study, the RF model we built for predicting CRC and the normal still returned a good performance with AUC of 0.835. Our model outperformed or were comparable with results in two recently published meta-analyses based on both 16S rRNA sequencing with smaller sample size (Shah et al., 2018) and metagenomic data (Dai et al., 2018), as well as some independent studies based on microbiota (Zeller et al., 2014; Baxter et al., 2016; Flemer et al., 2018) and other non-invasive procedures (FOBT and fecal Immunological test) (Zeller et al., 2014; Liang et al., 2017). Unexpectedly, the models for predicting adv_adenoma or adenoma from the normal were poor, which is consistent with results in the previous meta-analysis studies (Shah et al., 2018; Sze and Schloss, 2018). However, some studies did report better prediction for adenoma (Goedert et al., 2015; Baxter et al., 2016; Hale et al., 2017). Two potential reasons might explain the inconsistence between results from meta-analysis and the independent studies. Usually samples included in individual studies met consistent criterions, were treated by the same experimental and optimal analyzing protocols, and could be analyzed with more clinical data (e.g., FIT) to improve the model performances (Baxter et al., 2016). In contrast, there were great variations in these aspectsin the meta-analysis. Besides, the study number and sample size in our meta-analysis for adv_adenoma and adenoma were limited. Therefore, we are looking forward to more studies on adenoma to validate the potential of fecal bacteria in classifying adenoma from the individual with normal colon.

We also found that the RF model constructed using the common genera performed comparably with models based on the entire communities of total genera and even total OTUs, which means the fine level (OTU at 97% similarity) did not further improve the classification model. This phenomenon was also reported in a previous meta-analysis (Sze and Schloss, 2018) and individual study (Hannigan et al., 2018). The “patchy” hypothesis can be used to explain it (Sze and Schloss, 2018). As microbial distribution between individuals was patchy, the classification based on common genera will pool the fine-level diversity, and reduce the variations in the microbial features. Finally, Twelve common genera were identified as the most important features for distinguishing the CRC and the normal colon, 4 of which possessed significant ORs. Fusobacterium, one of the most frequently reported bacteria in CRC studies (Rubinstein et al., 2013; Yu et al., 2017), was enriched in CRC case relative control, as well as other pernicious genera, including *Escherichia _Shigella, Streptococcus.* We also identified the depletion of potentially beneficial microbes, such as the butyrate-producting *Anaerostipes Faecalibacterium, Lachnospira, Coprococcus* (Rivière et al., 2016; Vital et al., 2017). These genera could also be used for further validation by qPCR for more efficient diagnosis.

Even with best efforts, there were limitations in this study. We did not conduct further analyses to improve the RF model and for more subgroups, since we were unable to collect sufficient information regarding demographic data (age, gender, BMI etc.) and clinical data (FIT, FOBT, cancer stage, tumor location, adenoma growth patterns etc). Given this, we appeal researchers to share their sequencing and meta data associated to profoundly facilitate the research with larger sample size and more complete meta information (Quince et al., 2017). Moreover, it is expected to make better RF models for early screening and diagnosis by considering both microbial features and other metadata (including clinical data) (Baxter et al., 2016; Liang et al., 2017). An advantage in this study was that we obtained the tumor size, and tried to split adenoma samples into small adenoma and advanced adenoma, which was not provided in the previous meta-analyses.

In summary, our study uniformly analyzed a diverse collection of fecal 16S rDNA sequencing datasets and suggests the strong association between fecal bacterial community and colorectal tumors. By revealing the significant differences in diversity, identifying key taxa, and building RF model, we provide evidence for the use of fecal bacterial biomarkers to development of non-invasive diagnostic methods for the colorectal tumors, especially the CRC.

## Author Contributions

BZ, HX, and JR designed this study. BZ, SX, WX, QC, ZC, CY, YF, HZ, QL, JieY, JinY, and CX collected and organized the data. BZ, SX, WX, and HZ analyzed the data. BZ, SX, and WX wrote the manuscript.

## Conflict of Interest Statement

SX and HZ were employed by company Xiamen Treatgut Biotechnology Co., Ltd. The remaining authors declare that the research was conducted in the absence of any commercial or financial relationships that could be construed as a potential conflict of interest.
