# Supplementary figures and images for: Leveraging Fecal Bacterial Survey Data to Predict Colorectal Tumors
Source: Front Genet. 2019 May 28;10:447. doi: 10.3389/fgene.2019.00447 (PMC6547015; doi:10.3389/fgene.2019.00447)

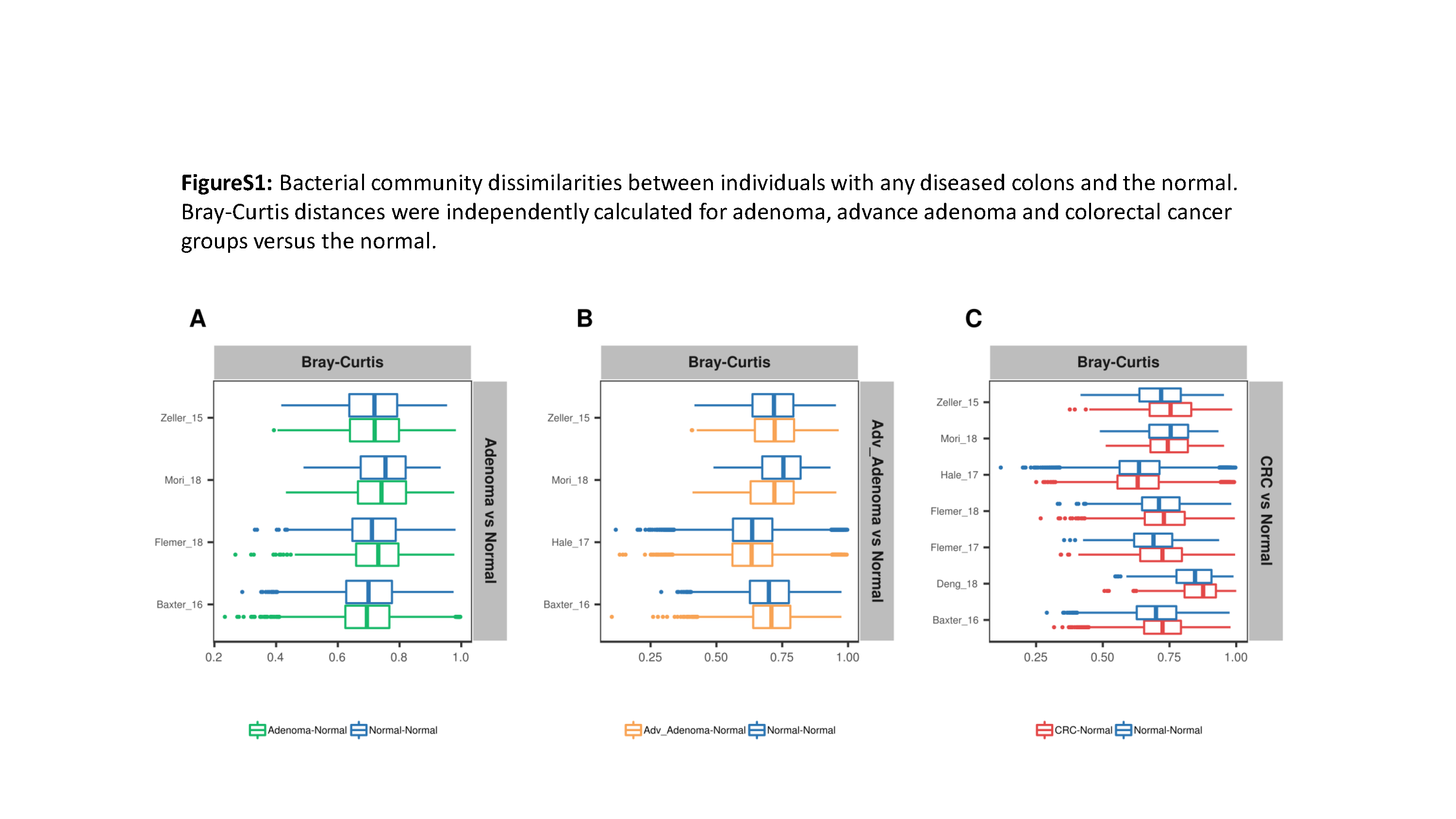

Supplement: Supplementary file 1 [file Image_1.TIFF]

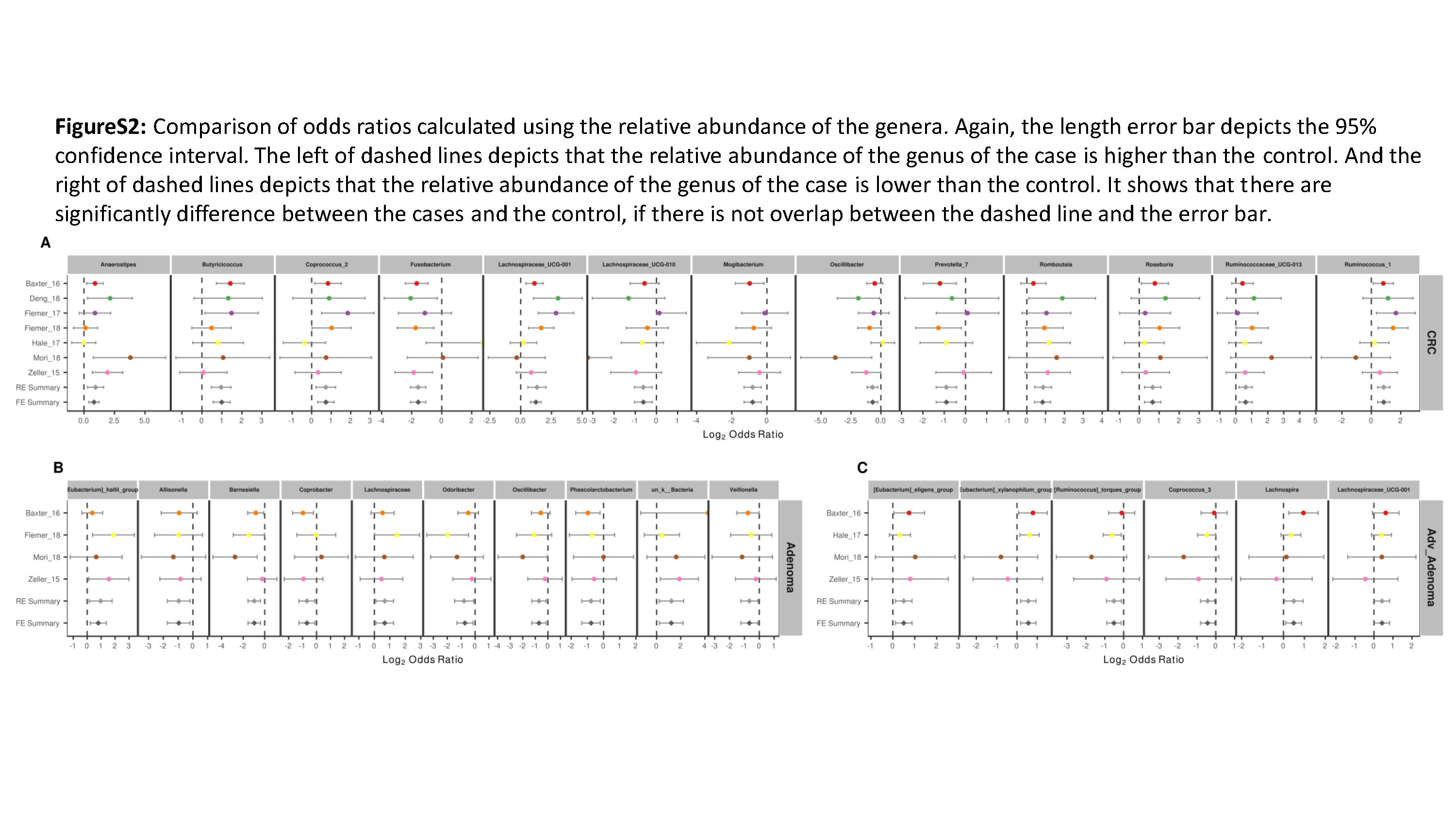

Supplement: Supplementary file 2 [file Image_2.TIFF]
